# Sequential backbone chemical shift assignments of a cancer-associated isoform of the HBx protein from human hepatitis B virus

**DOI:** 10.1007/s12104-025-10255-0

**Published:** 2025-11-08

**Authors:** Alexis Clavier, Toshinobu Shida, Maxim A. Droemer, Julian Holzinger, Anne K. Schütz

**Affiliations:** Faculty for Chemistry and Pharmacy, Ludwig-Maximilians-Universität München (LMU), 81377 München, Germany

**Keywords:** Hepatitis B virus (HBV), HBx protein, Solution NMR, NMR assignment, Automated assignment, ARTINA, Non-structural viral proteins

## Abstract

Chronic infections with hepatitis B virus (HBV) are a leading cause of liver cirrhosis and hepatocellular carcinoma worldwide. Among the four viral proteins encoded by HBV, the X protein (HBx) has remained resistant to atomic-level characterization. HBx is a small, non-structural protein that interacts with various human host proteins. It is essential for HBV replication and implicated in HBV-induced carcinogenesis. Here, we present the sequential backbone resonance assignments of a C-terminally truncated HBx isoform (residues 1–120), which is frequently found in chronically infected patients. Three-dimensional NMR experiments were recorded in the presence of residual urea (1 M), and the assignments of amide moieties were subsequently transferred to a low-urea condition (< 0.2 M) compatible with HBx interaction studies. We compare manual assignments with automated predictions using the ARTINA software. These results reveal secondary structure propensities in the truncated HBx isoform and lay the groundwork for future NMR-based studies of HBx interactions in solution.

## Biological context

Hepatitis B is one of the most common infectious diseases. It is caused by the small, enveloped Hepatitis B virus (HBV), which infects hepatocytes, the primary cells in the human liver. HBV infections can be acute or chronic (Tsukuda and Watashi 2020). It is estimated that one third of the world population has been acutely infected during their lifetime, of whom 3–4% remain chronically infected (WHO 2024). Such persistent infection can lead to the severe symptoms such as liver failure and cirrhosis. Moreover, hepatocellular carcinoma (HCC), which constitutes the third most common cause of cancer death worldwide (Siegel et al. 2024), is strongly associated with chronic HBV infection (Rizzo et al. 2022). Although a vaccine has been available since the 1980s, current antiviral strategies can only stop the virus from replicating without clearance (Fanning et al. 2019). Therefore, chronic HBV infection is still considered incurable (Than et al. 2019). 

The 3,200-base-pair DNA genome of HBV encodes four viral proteins: core, envelope, polymerase and the little-characterized X protein (HBx), which contains 154 amino acid residues (Tsukuda and Watashi 2020). HBx is essential for efficient replication of the viral genome, the covalently closed circular DNA (cccDNA) inside the nucleus of infected hepatocytes (Kornyeyev et al. 2019). HBx also localizes to the cytoplasm, where it associates with mitochondria (Kornyeyev et al. 2019). Generally, HBx targets viral restriction factors, modulates cellular signaling pathways and acts on viral promoters (Slagle and Bouchard 2016). It is reported to engage numerous host proteins (Wu et al., 2010; Van Damme et al. 2021); however, only few functional interactions are firmly established so far (He et al. 2023), partially due to challenges with reconstituting HBx in vitro. 

HBx is prone to aggregation and is mostly found in the insoluble pellet fraction when over-expressed in bacteria and insect cells, rendering denaturants such as urea necessary for in vitro studies (Lee et al. 2012; Urban et al. 1997). Because full-length HBx is poorly soluble without large tags and cannot reach concentrations required for atomic-level techniques like NMR, in-depth structural studies have so far been deterred. 

During chronic HBV infection with HCC development, linear fragments of viral DNA can become integrated into the human genome (Ma et al. 2008). During integration, the X gene is partially deleted to yield C-terminally truncated HBx isoforms, specifically HBx_ 1−120_, which spans the N-terminal 120 residues (Zhang et al. 2021). As a result, HBx_1−120_, rather than full-length HBx is frequently found in chronically infected patients and reported to be sufficient for the development of HCC by promoting cell proliferation (Zhang et al. 2021). The molecular-level effects of the truncation and its role in HCC progression remain largely unexplored. We have recently shown that HBx_1−120_ has a better solubility than full-length HBx, which makes it a suitable target for solution NMR (Clavier et al. 2025). Notably, the HBx_1−120_ construct significantly extends earlier studies (Kusunoki et al. 2022), which investigated a peptide encompassing HBx_101−120_ in fusion and in complex with the Bcl-xL host protein. Previously, both N- and C-terminally truncated HBx variants harboring five cysteine to serine mutations were monitored in two-dimensional (2D) ^1^H-^15^N NMR correlation spectra, yet not assigned sequentially (Lee et al. 2012). In view of the putative zinc binding by cysteine residues in HBx (Ramakrishnan et al. 2019), it is desirable to work with the unmodified sequence. 

Here, we present the ^1^H, ^15^N and ^13^C sequential backbone resonance assignments for HBx_1−120_ from three-dimensional (3D) NMR correlation spectra recorded in the presence of 1 M urea. The sequential assignment is transferred to 2D ^1^H-^15^N amide correlation spectra of HBx_1−120_ in 190 mM urea and confirmed by a series of point mutants. The latter low-urea condition is compatible with assays to probe the interactions of HBx with host proteins (Clavier et al. 2025). We also evaluate the automated assignments generated by the ARTINA deep learning model. Our work paves the way for studies that examine the structural properties of HBx_1−120_ in solution and investigate the molecular basis of its many reported host protein interactions.

## Methods and experiments

### Protein expression and purification

The production of recombinant HBx_1−120_ in *Escherichia coli* (*E. coli*) and its analytical characterization are described in detail in (Clavier et al. 2025) and briefly summarized below.

The HBx_1−120_ sequence was cloned into the pET28a(+) expression vector, from which the N-terminal hexa-histidine tag sequence was removed to obtain untagged HBx_1−120_. The sequence corresponds to the HBV genotype D (Uniprot P03165, natural variant R26C). Point mutations were introduced by Q5 site-directed mutagenesis (New England Biolabs, Ipswich, MA, USA) with custom-designed primers. These mutations were D48G, H49G, V60G, S65G, M79G, E80G, N84G, Q87G, H94G, K95G, M103G and L108G. Each mutation was verified by sequencing.

All HBx_1−120_ proteins were overexpressed in *E. coli* BL21(DE3) cells in M9 medium containing ^15^NH_4_Cl and ^13^C glucose as the sole source of isotopically labelled nitrogen (0.5 g/L) (Merck, Darmstadt, German) and carbon (3 g/L) (Merck, Darmstadt, German), respectively, with expression induced at OD_600_ ~ 0.6–0.8 at 37 °C for 4 h.

The cells were lysed by sonication in 50 mM Tris-HCl, pH 8.0, 150 mM NaCl, 0.5 mM EDTA, 1 mg/ml lysozyme with protease inhibitor and centrifuged. The pellet was then washed with lysis buffer plus 0.1% Triton X-100, centrifuged and resuspended in solubilization buffer (50 mM Tris-HCl, pH 8.0, 500 mM NaCl, 8 M urea, 5 mM imidazole and 5mM DTT). The suspension was incubated for at least 48 h at 4 °C to dissolve HBx_1−120_ from inclusion bodies, centrifuged and the supernatant dialyzed into cation exchange buffer A (50 mM MES, pH 6.0, 6 M urea and 5 mM DTT). The dialyzed sample was applied to a HiTrap SP HP cation-exchange chromatography column (Cytiva, Marlborough, MA, USA) and HBx_1−120_ was eluted in a 0–50% gradient of buffer supplemented with 1 M NaCl. The eluted fractions were further purified by size-exclusion chromatography (SEC, HiLoad Superdex 200 pg, Cytiva) in 50 mM HEPES, pH 7.4, 50 mM NaCl, 6 M urea and 5 mM DTT.

For the preparation of the high-molar urea sample, HBx_1−120_ was dialyzed in a stepwise manner from SEC buffer to *(1)* 4 M urea, 0.5 M L-arginine, 150 mM NaCl, 50 mM HEPES pH 7.4, 5 mM DTT, followed by *(2)* 2 M urea, 0.25 M arginine, 150 mM NaCl, 50 mM HEPES pH 7.4, 5 mM DTT and finally into *(3)* 1 M urea, 0.125 M arginine, 150 mM NaCl, 50 mM HEPES, 5mM DTT, pH 7.4. The protein was concentrated to approximately 300 µM with a 3 kDa MWCO Amicon ultracentrifugal filter unit (Merck Millipore, Burlington, MA, USA) at 4 °C. The low-molar urea sample was dialyzed from the SEC buffer to *(1)* 4 M urea, 1 M arginine, 150 mM NaCl, 50 mM HEPES pH 7.4, 5mM DTT followed by a dialysis to the buffer *(2)* 1.5 M urea, 1 M arginine, 150 mM NaCl, 50 mM HEPES pH 7.4, 5 mM DTT. After having been concentrated to 400 µM, the HBx_1−120_ protein was rapidly diluted eightfold in the buffer *(3)* 150 mM NaCl, 50 mM HEPES pH 7.4 to reach the concentration of 50 µM in the final buffer 0.19 M urea, 0.125 M arginine, 150 mM NaCl, 50 mM HEPES pH 7.4, 0.62 mM DTT.

## NMR spectroscopy

Solution NMR spectra were acquired at 4 °C on a Bruker Avance III 800 MHz spectrometer (18.8 T), using a cryogen-cooled TCI probe. All spectra were processed with Topspin version 3.7.0 (Bruker, Billerica, MA, USA) and analyzed using CcpNmr program version 3.2.2 (Skinner et al. 2016). Non-uniformly sampled (NUS) experiments used a Poisson gap sampling schedule (Robson et al. 2019) and were reconstructed with NMRPipe (Delaglio et al. 1995) using the SMILE algorithm (Ying et al. 2017). ^1^H^15^N^13^C-HNCACB and HNCO NMR experiments were recorded in 1 M urea, 0.125 M arginine, 150 mM NaCl, 50 mM HEPES pH 7.4, 5 mM DTT at a concentration of 280 µM in a 5 mm NMR tube (Deutero, Kastellaun, Germany). The ^1^H^15^N^15^N-HN(CA)NNH NMR (HNN) experiment was recorded in a different buffer (2 M urea, 0.25 M arginine, 150 mM NaCl, 50 mM HEPES pH 7.4, 5 mM DTT) at a concentration of 300 µM in a 5 mm Shigemi tube (Shigemi, Tokyo, Japan). The reason for recording the HNN experiment in the presence of higher concentrations of urea and arginine was the increased stability of HBx_1−120_ under these conditions. The information obtained from the HNN spectrum can nevertheless be transferred to the other 3D spectra as the resonance positions are still relatively similar. The HNN NMR spectrum was used to complement and validate the sequential walk based on the HNCACB spectrum. The ^1^H-^15^N SOFAST-HMQC NMR experiments were recorded in the low-urea condition (0.19 M urea, 0.125 M arginine, 150 mM NaCl, 50 mM HEPES pH 7.4, 0.62 mM DTT) at a concentration of 50 µM in a 5 mm NMR tube. The same buffer conditions and NMR acquisition parameter were used for the mutant HBx_1−120_ proteins. All measurement parameters are summarized in Table 1.

**Table 1:** Overview of experimental parameters for all NMR spectra used for the sequential resonance assignment of HBx_1−120_

Sample buffer	Protein concentration	Labelling	Experiment	Points	NUS %	NS	Sweep widths (ppm)	Acquisition times (ms)	Carrier frequencies (ppm)
1 M urea, 125 mM arg.	270 M	^15^N - ^13^C	Best_HNCACB (NMRlib) (Favier and Brutscher 2019)	2048/128/280 (H-N-C)	10.0	160	14.039/27/80 (H-N-C)	91.1/29.2/8.69 (H-N-C)	4.7/118/39 (H-N-C)
1 M urea, 125 mM arg.	270 M	^15^N - ^13^C	Best_HNCO (NMRlib) (Favier and Brutscher 2019)	2048/192/96 (H-N-C)	10.0	96	12.06/27/15 (H-N-C)	106.0/43.8/15.9 (H-N-C)	4.7/118/173.5 (H-N-C)
2 M urea, 250 mM arg.	300 M	^15^N - ^13^C	hncannhgpwg3d (Bruker library) (Weisemann et al. 1993)	2048/140/140 (H-N-N)	10.0	192	16.02/23/23 (H-N-N)	79.9/37.5/37.5 (H-N-N)	4.7/118.5/118.5 (H-N-N)
1 M urea, 125 mM arg.	270 M	^15^N - ^13^C	sfhmqcf3gpph (Bruker library) (Schanda et al. 2005)	2048/512 (H-N)	-	32	16.02/31.99 (H-N)	79.9/98.6 (H-N)	4.7/120 (H-N)
190 mM urea, 125 mM arg.	50 M	^15^N	sfhmqcf3gpph (Bruker library) (Schanda et al. 2005)	2048/512 (H-N)	-	256	16.02/31.99 (H-N)	79.9/98.6 (H-N)	4.71/120 (H-N)

L-arginine is abbreviated as arg. All experiments were carried out at a **¹**H resonance frequency of 800 MHz at 4 °C using a cryogen-cooled triple-resonance probe

Automated chemical shift assignments were performed using ARTINA (Klukowski et al. 2022). The built-in automated peak picking function was applied on the HNCACB and HNCO spectra. Secondary structure prediction and the random coil index order parameter (RCI-S^2^) calculation was performed using the TALOS-N server (Shen and Bax 2015) employing the manually assigned chemical shifts.

## Extent of assignments and data deposition

**Fig. 1:**
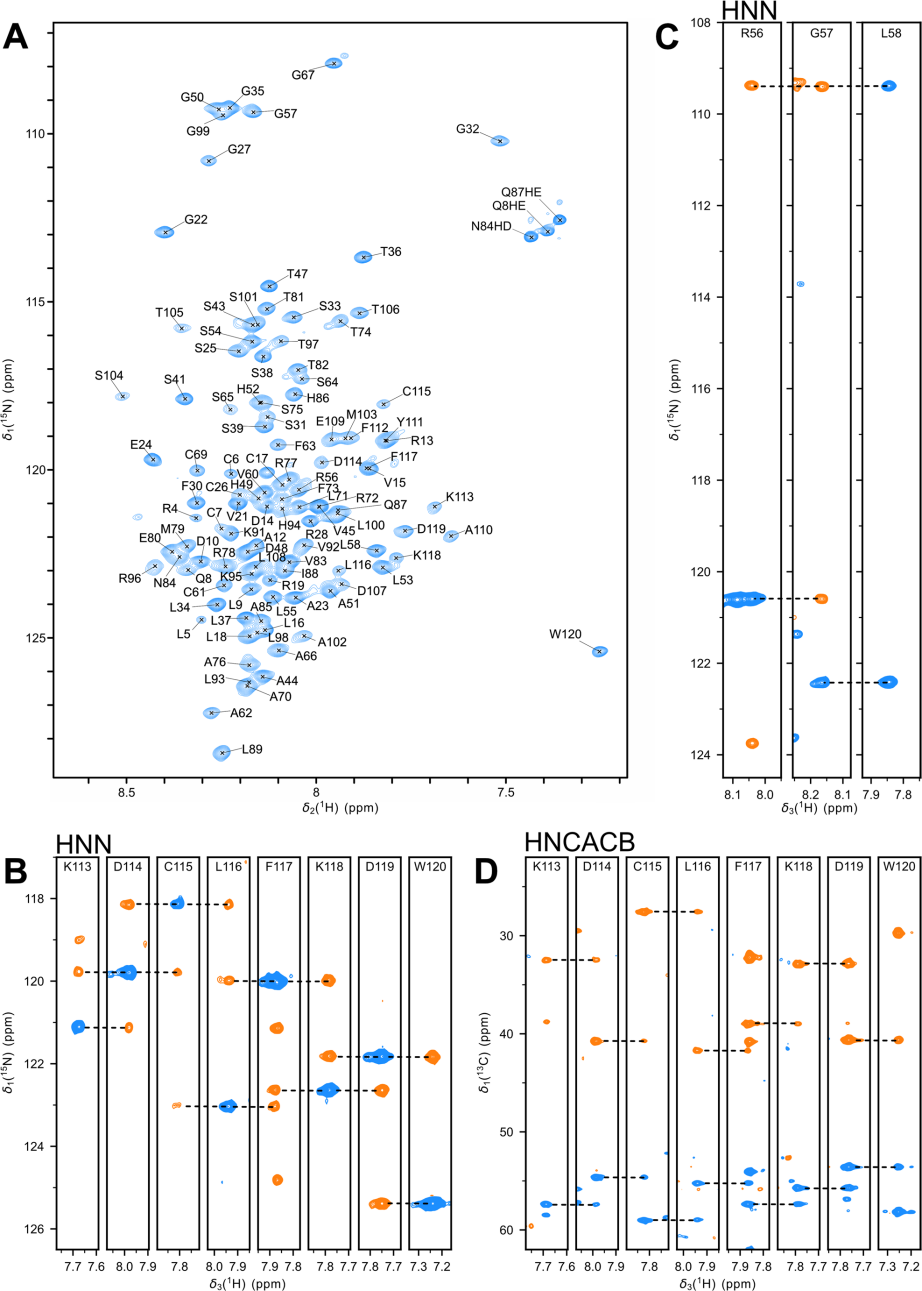
**(A)** ^1^H-^15^N SOFAST-HMQC spectrum of HBx_1–120_ in 1 M urea, 125 mM L-arginine, 150 mM NaCl, 50 mM HEPES pH 7.4 at 4 °C. **(B-D)**. Representative strip plots from 3D assignment spectra. Positive contours in blue, negative in orange. **(B)** Strips from the HNN spectrum documenting the sequential walk between neighboring residues starting from the C-terminus. **(C)** Strips from the HNN spectrum highlighting the variations in the phase patterns (positive/negative) for residues neighboring proline and glycine. **(D)** Strips from the HNCACB spectrum for the sequential walk starting from the C-terminus

The fully assigned ^1^H-^15^N SOFAST-HMQC amide correlation spectrum of HBx_1−120_ recorded in presence of 1 M urea is shown in Fig. 1A. Since HBx_1−120_ contains 120 amino-acid residues including 9 proline residues, 111 cross peaks are expected in this spectrum. Overall, backbone amide correlations of 108 residues could be assigned, which corresponds to 97.3%. Of the expected ^13^C resonances 96.7% ^13^C^α^, 97.3 ^13^C^β^ and 97.3% ^13^CO could be assigned. The missing assignments correspond to the N-terminal residues M1, A2 and A3, which are not detectable. Compared to a folded protein, HBx_1−120_ shows a narrow chemical shift dispersion in the ^1^H dimension, strongly suggesting that it is predominantly disordered. Subsequently, all amide correlations were transferred to the 190 mM urea buffer condition. The sequential backbone chemical shift assignments (amide ^1^H and ^15^N and backbone ^13^C^α^, ^13^C^β^, ^13^CO) of HBx_1−120_in 1 M urea and the ^1^H and ^15^N amide assignment in 190 mM urea were deposited to the Biological Magnetic Resonance Bank (BMRB) under the accession number 53376.

The low chemical shift dispersion in the ^1^H dimension raises the risk of signal overlaps, potentially resulting in missed or erroneous assignments. Therefore, a 3D HNN spectrum was additionally recorded to correlate the ^1^H-^15^N amide resonance pairs NH_*i*_ of a given amino-acid residue *i* with the ^15^N resonances N_*i*−1_ and N_*i*+1_ of its neighboring residues (Panchal et al. 2001). Within the strip corresponding to a given NH_*i*_ pair, correlations to neighboring residues N_*i*−1_ and N_*i*+1_ exhibit opposite phase relative to the N_*i*_ correlation (Fig. 1B). The HNN spectrum is complementary to the HNCACB spectrum, whose conventional strip plots highlight the sequential walk along the aliphatic resonances C^α^ and C^β^ (Fig. 1D). Moreover, the HNN spectrum pinpoints residues neighboring a proline residue, for which sequential correlations are absent. This is exemplified in the strip of residue L58 preceding P59, showing only N_*i*_ and N_*i*−1_ correlations (Fig. 1C). Furthermore, for glycine residues, the N_*i*_ and N_*i*−1_ correlations will be in the same phase, and the N_*i*+1_ correlation will be in the opposite phase. This specific pattern defines the direction of the sequential walk, the strip of G57 is shown as an example (Fig. 1C). Finally, the residue following the glycine will also show a distinct phase pattern as its N_*i*_ and N_*i*−1_ correlations will be in the same phase, which helps to detect residues neighboring glycine, as illustrated for L58 following G57 (Fig. 1C).

**Fig. 2:**
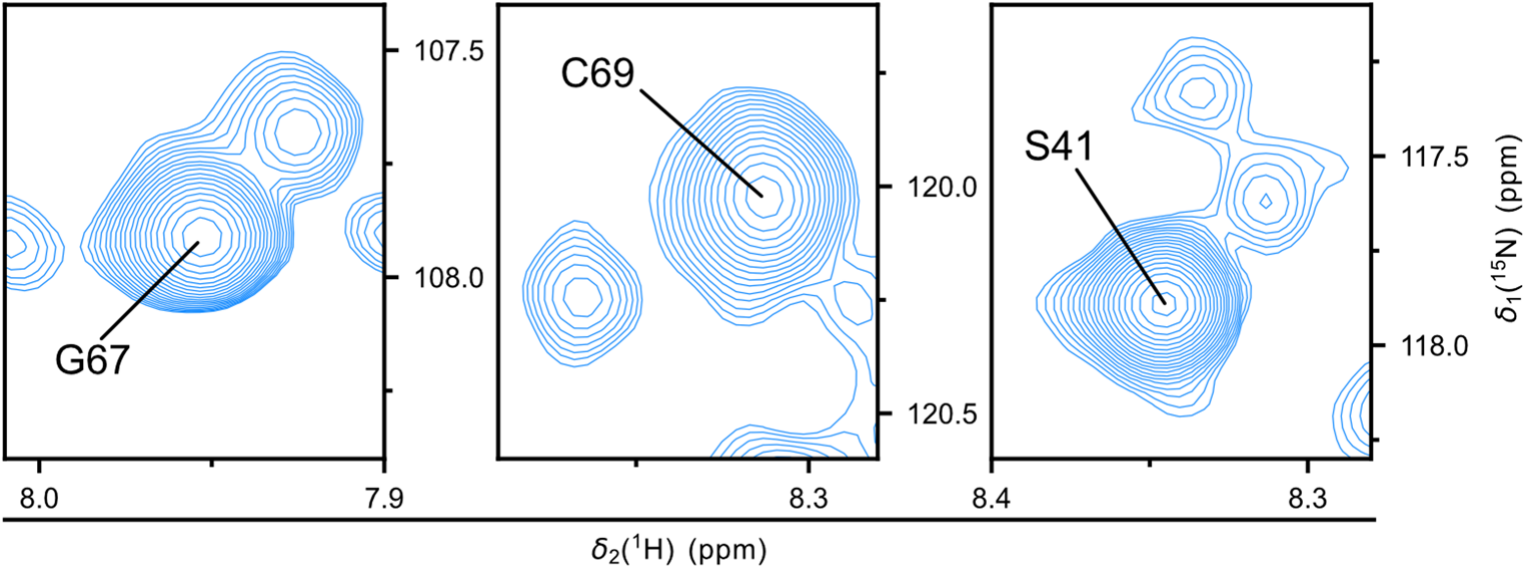
Excerpts from ^1^H-^15^N SOFAST-HMQC spectrum of HBx_1−120_ in 1 M urea (same spectrum as Fig. 1A, here visualized at lower contour level) that highlight residues neighboring prolines. The amide correlations display a peak splitting that might represent a mixture of proline cis and trans isomers in slow exchange. As an example, satellite peaks of G67 and C69 are shown, adjacent to P68. The signal assigned to S41, which is located in between the two prolines P40 and P42, exhibits several satellite peaks. These split signals are picked by ARTINA as measured peaks but remained unassigned by the software

**Fig. 3:**
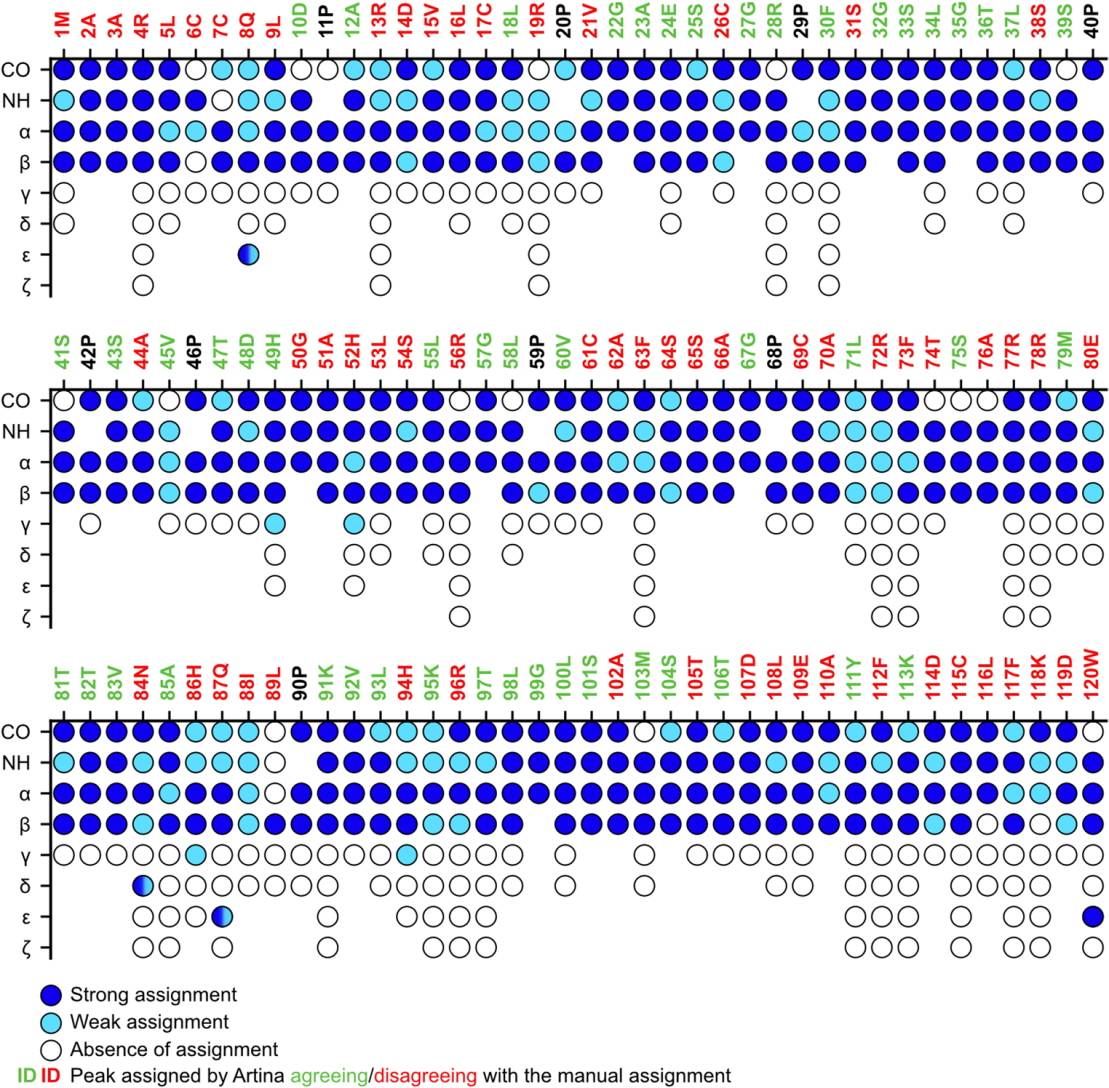
FLYA (fully automated assignment) (Schmidt and Güntert 2012) plot showing automated resonance assignments for the HBx_1–120_ sequence. Each residue is represented along the x-axis, with assignment confidences for different atom types (HN, CO, C^α^, C^β^ and side chain) indicated as blue for high confidence, cyan for low confidence and white for unassigned. Residues for which automatic and manual assignment match are indicated in green, disagreements in red, and non-detectable proline residues in black

In parallel to the manual assignment, we employed ARTINA (Klukowski et al. 2022), a machine-learning-based automated assignment model, and compared its results with our manual assignments for HBx_1−120_ (Table 2). ARTINA was given the SOFAST-HMQC, HNCACB and HNCO spectra as input for the automated peak picking and assignment. The HNN spectrum was not provided as input in ARTINA because this more specialized experiment is less represented in the program training dataset; moreover, minor chemical shift changes due to the different buffer could mislead the software. The self-assessed accuracy of ARTINA was estimated at 76% of assigned residues within the protein backbone (92 out of 120). However, ARTINA overestimated the number of peaks in every spectrum, with detected peaks exceeding the expected count. Consequently, ARTINA tried to assign spectral noise as peaks (Table 2) and satellite peaks, which could reflect a mixture of cis and trans isomers of residues neighboring proline (Fig. 2). When comparing our manual assignments to the ARTINA output, we found that the prediction was correct in 44% of the cases, with 49/111 peaks matching the manual assignment (Fig. 3). As ARTINA was trained largely on folded proteins, it is anticipated that its application to a disordered protein like HBx_1−120_ will be less effective (Klukowski et al. 2024). Furthermore, ARTINA seems to be challenged by assignments of residues neighboring proline, for which information confirming C^α^ and C^β^ chemicals shifts is absent. Nevertheless, weighing the useful starting points for the assignment provided by ARTINA compared to the little time invested to prepare the input files, ARTINA still greatly contributes to facilitating the time-consuming task of biomolecular NMR assignment.

**Table 2:** Results of the automated sequential resonance assignment by ARTINA

Spectrum	Expected	Assigned expected	Measured	Assigned measured
HNCACB	425	377 (88.71%)	476	331 (69.54%)
HNCO	110	95 (86.36%)	126	86 (68.25%)
SOFAST-HMQC	131	119 (90.84%)	169	111 (65.68%)

Expected: Number of peaks expected in the spectrum if all non-proline residues of HBx₁₋₁₂₀ were fully detectable. Assigned expected: Number of expected peaks that are assigned to a detected (automatically picked) peak. The percentage is relative to the number of expected peaks. Measured: Number of detected (automatically picked) peaks. Assigned measured: Number of measured peaks that have one or more expected peaks assigned to it. The percentage is relative to the number of measured peaks

**Fig. 4:**
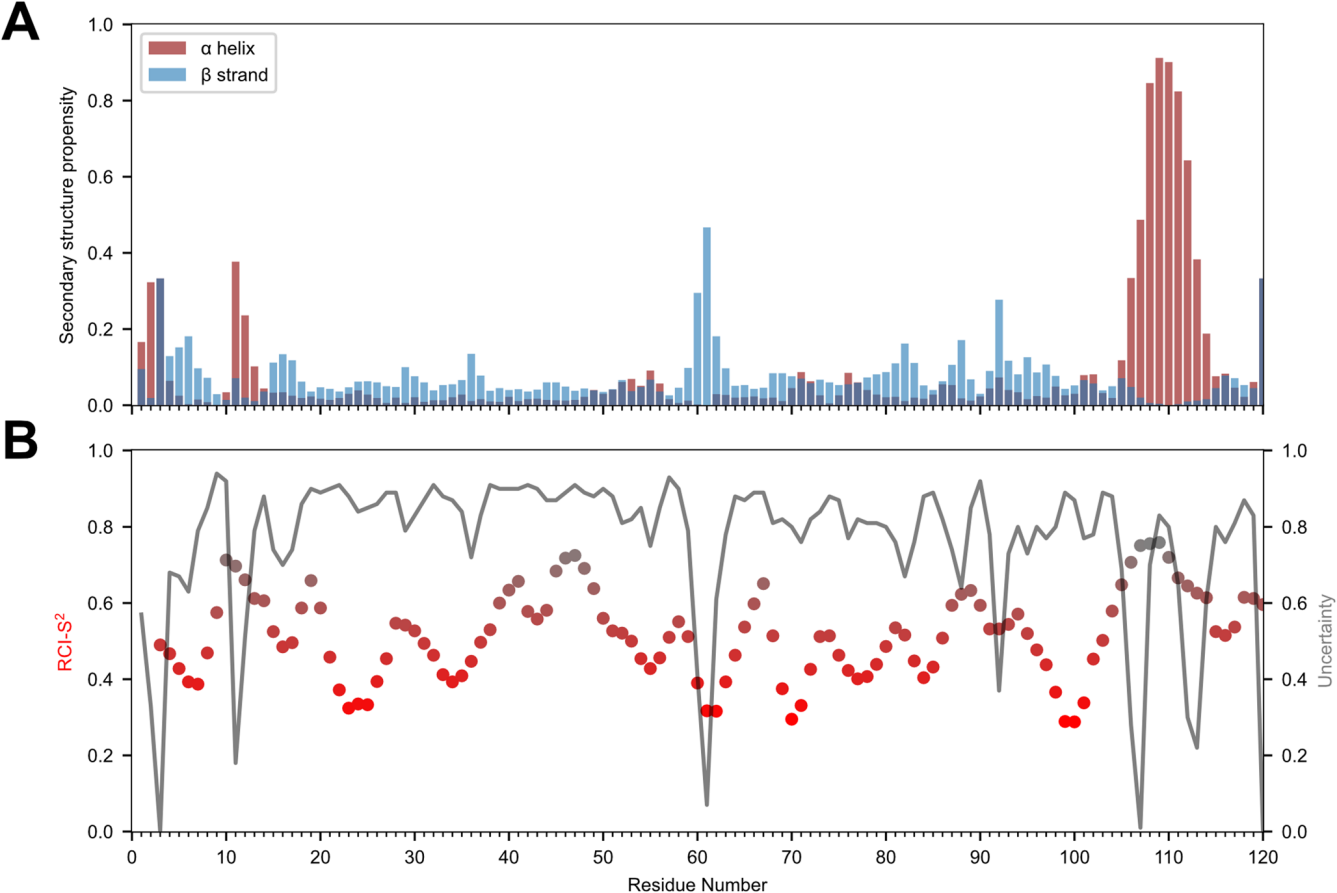
TALOS-N (Shen and Bax, 2015) predictions showing for each residue **(A)**. the secondary structure propensity and **(B)**. the predicted backbone rigidity reflected in the random coil index order parameter RCI-S², which scales between 0 (total disorder) and 1 (fully rigid). Residues below the threshold RCI-S² ≤ 0.6 are assigned as dynamic (Shen and Bax, 2015). The uncertainty represents the confidence of the three-state (α-helix, β-strand, loop) secondary structure prediction for a given target residue. (Results were plotted thanks to the script of Prof. Woonghee Lee.)

We further analyzed the secondary structure of HBx_1−120_ in 1 M urea using TALOS-N (Shen and Bax 2015). TALOS-N received all chemical shifts obtained from the HNCACB and HNCO spectra as an input. The presence of only one well-defined secondary structural element was detected in the 1 M urea condition (Fig. 4A). Specifically, residues 105–117 show high α-helical propensity, suggesting that in 1 M urea, HBx_1−120_ is not fully disordered, yet still predominantly possesses the characteristics of an intrinsically disordered protein. We further analyzed random coil index order parameter RCI-S^2^ (Fig. 4B). The closer this index gets to zero, the more flexible is the protein region. Residues below the threshold RCI-S^2^ ≤ 0.6 TALOS-N assigns as dynamic, which is the case for most of the protein sequence, coherent with the disordered propensity of the HBx_1−120_ protein. The residual 1 M urea, in which the sequential NMR assignment was conducted, could still have a denaturing effect on the HBx_1−120_. Therefore, we also evaluated a second condition that is closer to the native one.

**Fig. 5:**
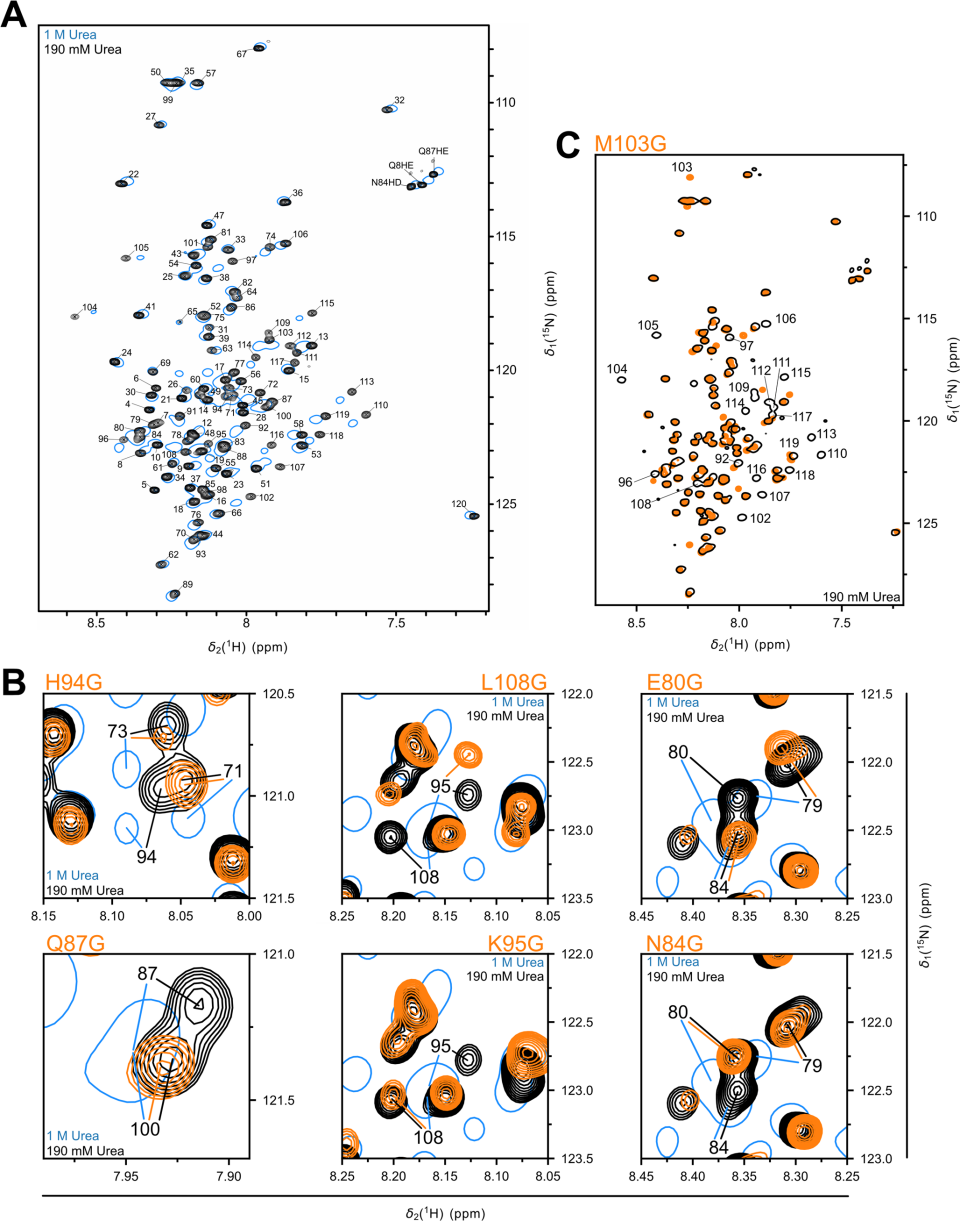
**(A)** Overlay of the ^1^H-^15^N SOFAST-HMQC spectra of HBx_1−120_ in high-urea condition (blue single contours) and low-urea condition (black contours), including the sequential assignment. The assignments were manually transferred from the first to the second condition. **(B)** Spectra of twelve point mutants were recorded to resolve ambiguous assignment transfers for signals overlapping in the high-urea condition. Excerpts from six point mutants are overlaid with the wild-type spectra. **(C)** Overlay of spectra recorded of wild type and M103G HBx_1−120_ in the low-urea condition. Out of twelve mutations tested, only M103G and L108G showed a significant allosteric effect. Glycine insertion in the C-terminal BH3-like helix caused major chemical shift perturbations affecting ± 15 residues around the mutation site, revealing a cooperative disruption of the secondary structure

We lowered the urea concentration to 190 mM and recorded a ^1^H-^15^N SOFAST-HMQC spectrum to obtain sequential NMR assignments under these more physiological conditions (Fig. 5A). HBx_1−120_ remains soluble despite its strong tendency to aggregate. Notably, the overall spectral changes are subtle and the dispersion in the ^1^H dimension remains low in 190 mM urea. We conclude that the protein does not fold even at urea levels so low that they are not expected to counteract secondary structure formation in folded proteins (Nick Pace et al. 2010). Overall, the HMQC spectra in 1 M and 190 mM urea superimpose well. A sequential assignment from 3D spectra of the low-urea sample could not be accomplished since this condition does not allow a protein concentration higher than 50 µM. Instead, all assigned peaks were manually transferred from the high-urea to the low-urea condition (Fig. 5A). Point mutants of HBx_1−120_ were utilized to resolve ambiguities appearing during this transfer and to confirm the assignments. Figure 5B shows six representative spectral overlays, out of 12 obtained, comparing wild-type and mutant HBx_1−120_. While most point mutations introduced only local chemical shift perturbations to the adjacent two or three residues, more extensive perturbations were caused by the mutation M103G (Fig. 5C), consistent with a larger cooperative effect on the whole C-terminal region of the protein. A similar effect is observed for L108G. The TALOS-N-predicted helix in this region may account for the stronger effects of mutations at positions 103 and 108, by affecting the formation or stability of the helix. These effects precluded a definite assignment of residues M103 and E109 by mutagenesis, which cannot be unambiguously transferred from the high to the low urea condition. TALOS-N was not repeated for the low-urea condition due to the absence of ^13^C shifts, which are central to secondary structure prediction. The transferred sequential backbone chemical shift assignments (amide ^1^H and ^15^N) of HBx_1−120_ in the low urea condition have been deposited in in the BMRB under the accession number 53376.

## Conclusion

We report the nearly complete sequential backbone chemical shift assignments of a C-terminally truncated HBx isoform, HBx_1–120_, under mildly denaturing conditions. These assignments were shown to be reliably transferrable from a high-urea (1 M) to a low-urea (190 mM) environment, the latter being well-suited for functional studies such as binding experiments. Comparison with automated assignments generated by ARTINA highlights the potential and current limitations of deep learning-based strategies for analyzing NMR spectra of intrinsically disordered proteins. This assignment provides a foundation for NMR-based studies of HBx structural dynamics and interactions. These studies will aim at unveiling the molecular mechanisms by which HBx contributes to HBV pathogenesis, particularly the role of HBx isoforms in the development of hepatocellular carcinoma.

## Data Availability

The ^1^H, ^15^N and ^13^C sequential backbone (^1^H, ^15^N, ^13^C^α^, ^13^C^β^, ^13^CO) resonance assignments for HBx_1–120_ in 1 M urea and the transferred assignment (^1^H, ^15^N) for HBx_1–120_ in 190 mM urea have been deposited in the BMRB under the accession number 53376. All other data presented in this study are available upon request.
